# Dynamics of flavonoid metabolites in coconut water based on metabolomics perspective

**DOI:** 10.3389/fpls.2024.1468858

**Published:** 2024-10-07

**Authors:** Mingming Hou, Jerome Jeyakumar John Martin, Yuqiao Song, Qi Wang, Hongxing Cao, Wenrao Li, Chengxu Sun

**Affiliations:** ^1^ School of Life Sciences, Henan University, Kaifeng, Henan, China; ^2^ Coconut Research Institute, Chinese Academy of Tropical Agricultural Sciences, Wenchang, China; ^3^ National Key Laboratory for Tropical Crop Breeding, Chinese Academy of Tropical Agricultural Sciences, Haikou, China; ^4^ College of Wine and Horticulture, Ningxia University, Yinchuan, Ningxia Hui Autonomous Region, China

**Keywords:** coconut water, flavonoid metabolites, developmental dynamics, liquid chromatography tandem mass spectrometry, metabolomics

## Abstract

Coconut meat and coconut water have garnered significant attention for their richness in healthful flavonoids. However, the dynamics of flavonoid metabolites in coconut water during different developmental stages remain poorly understood. This study employed the metabolomics approach using liquid chromatography-tandem mass spectrometry (LC-MS/MS) to investigate the changes in flavonoid metabolite profiles in coconut water from two varieties, ‘Wenye No.5’(W5) and Hainan local coconut (CK), across six developmental stages. The results showed that a total of 123 flavonoid metabolites including chalcones, dihydroflavonoids, dihydroflavonols, flavonoids, flavonols, flavonoid carboglycosides, and flavanols were identified in the coconut water as compared to the control. The total flavonoid content in both types of coconut water exhibited a decreasing trend with developmental progression, but the total flavonoid content in CK was significantly higher than that in W5. The number of flavonoid metabolites that differed significantly between the W5 and CK groups at different developmental stages were 74, 74, 60, 92, 40 and 54, respectively. KEGG pathway analysis revealed 38 differential metabolites involved in key pathways for flavonoid biosynthesis and secondary metabolite biosynthesis. This study provides new insights into the dynamics of flavonoid metabolites in coconut water and highlights the potential for selecting and breeding high-quality coconuts with enhanced flavonoid content. The findings have implications for the development of coconut-based products with improved nutritional and functional properties.

## 1 Introduction

Coconut is an important tropical cash crop known for its nutrient-rich meat and water. Coconut water in particular, is a popular natural beverage packed with essential nutrients (Pummer et al., 2001). Coconut water is reported to contain 5-9% total soluble solids (TSS), with over 80% being soluble sugars like glucose, sucrose and fructose (Mahayothee et al., 2016). Additionally, coconut water contains essential components including minerals, amino acids, enzymes, organic acids, fatty acids, vitamins and few phenolic compounds (Kumar et al., 2021). Phenolic compounds are recognized for their bioactive properties, with coconut water containing catechin, salicylic acid, 4-hydroxybenzoic acid, butyric acid, o-coumaric acid, p-coumaric acid, gallic acid and caffeic acid in coconut water (Chang and Wu, 2011; Mahayothee et al., 2016).

Flavonoids as a type of polyphenols widely found in many plants (Kozlowska and Szostak-Wegierek, 2014; Rudrapal and Chetia, 2016), are typically categorized into seven subclasses including, flavonols, flavones, isoflavones, anthocyanins, flavanones, flavanols, and chalcones (Wang et al., 2022). Research has demonstrated that flavonoids are prevalent in plants and play a crucial role in plant growth, reproduction and protection (Tang et al., 2014; Tian et al., 2014; Deng et al., 2019). Flavonoids serve as major pigments in plants and function as antitoxins or antioxidants, protecting plants from biotic and abiotic stress (Cavaiuolo et al., 2013; Iwashina, 2003; Zhang et al., 2020; Pourcel et al., 2007). In the fields of medicine and health, plant-derived flavonoids, as a bioactive compound, have been found to exhibit a broad spectrum of pharmacological effects, including neurological relief, anti-inflammatory, antioxidant, hepatoprotective (Leyva-López et al., 2016; Liu et al., 2017; Al-Khayri et al., 2022; Shen et al., 2022). Furthermore, flavonoids have been shown to help prevent osteoporosis, cancer, and reduces the risk of cardiovascular disease (Imran et al., 2019; Wang et al., 2017; Fernandes et al., 2017; Liu et al., 2021), ultimately having a profound impact on promoting human health and well-being (Tungmunnithum et al., 2018; Batiha et al., 2020; Kumar et al., 2017).

Metabolomics as a key histological technology that focuses on the precise qualitative and quantitative analysis of metabolites, and subsequently performs an in-depth analysis of metabolic pathways and network structures, providing a solid scientific foundation for revealing the metabolic mechanisms underlying the macroscopic phenotypes of different organisms (Fiehn, 2002). In particular, the integration of liquid chromatography-tandem mass spectrometry (LC-MS/MS) technology with chemoinformatics methods has significantly enhanced the ability to screen the diversity of complex metabolites, such as flavonoids, in a high-throughput manner (Akimoto et al., 2017; Tsugawa et al., 2016, 2019).

Coconut water, a natural beverage, that is rich in compounds, but there is a scarcity of reports on flavonoids. The change trend and metabolic mechanism of flavonoids in coconut water at different developmental stages are still unclear. To address this, we employed a combination of LC-MS/MS technique and metabolomics to conduct an in-depth study of flavonoid metabolites in coconut water at different developmental stages of the fruits of two coconut varieties. The aim of this study was to elucidate the characteristics of changes in flavonoid metabolites during the developmental stages of different coconuts and their roles during the ripening process of coconut fruits. The findings on flavonoid synthesis can offer insights into improving coconut quality. Understanding how flavonoids are synthesized in different coconut varieties can provide a scientific basis for enhancing their quality and nutritional value. This research may lead to better methods for heterologous synthesis of flavonoids, contributing to both theoretical knowledge and practical applications in coconut variety improvement.

## 2 Materials and methods

### 2.1 Experimental materials and treatments

The test materials used in this study were 10-year-old ‘Wenye No.5’ (W5) and 10-year-old local high species of coconut (CK). For each variety, three coconut trees were selected, and 100 mL of coconut water was collected from each fruit at six distinct developmental stages: 2, 4, 6, 8, 10, and 12 months of age. At each stage, three replicate samples of coconut water were collected in parallel. The collected coconut water was immediately flash-frozen in liquid nitrogen and stored at -80°C in a refrigerator for future analysis.

### 2.2 Determination and analysis of flavonoid metabolites

The combination of chromatography and mass spectrometry facilitates the whole process from separation of substances by chromatography to identification of substances by mass spectrometry. Flavonoids in coconut water can be accurately characterized and quantified using ultra-high performance liquid chromatography-mass spectrometry (UHPLC-MS/MS). The samples were thawed from -80°C refrigerator and rotated for 10 s to mix them thoroughly. The mixed 9 mL sample was placed in a 50 mL centrifuge tube and immersed in liquid nitrogen. After the sample was completely lyophilized, 300 uL of 70% methanol internal standard extraction solution was added and spun for 3 minutes. The mixture was then centrifuged at 12,000 rpm, 4°C for 10 min. The supernatant was filtered through a 0.22 μm microporous filtration membrane and stored in a sample vial for UPLC-MS/MS detection. Metabolite data obtained from mass spectrometry were preprocessed and quality controlled to ensure accurate quantification. Orthogonal partial least squares discriminant analysis (OPLS-DA) and differential multiple integration were used to screen for differential metabolites. The screening thresholds were VIP≥1,Fold_Change≥2 or Fold_Change ≤ 0.5 to identify differential metabolites.

### 2.3 Chromatography and Mass Spectrometry Acquisition Conditions

The data acquisition instrument system included Ultra Performance Liquid Chromatography (UPLC) (SHIMADZU Nexera X2) and Tandem mass spectrometry (Applied Biosystems 4500 QTRAP).

The liquid phase conditions mainly include:

1. Column: Agilent SB-C18 1.8µm, 2.1mm * 100mm;

2. Mobile phase: phase A is ultra-pure water with 0.1% formic acid; phase B is acetonitrile with 0.1% formic acid.

3. Elution gradient: The proportion of B phase is 5% at 0.00 minutes, increases linearly to 95% within 9.00 minutes, and is maintained at 95% 1 minute. From 10.00-11.10 minutes, the proportion of B phase decreases to 5%, and is balanced at 5% untill14 minutes.

4. Flow rate 0.35 mL/min; Column temperature: 40°C; Sample size: 4 μL.

The mass spectrometry conditions mainly include:

LIT and Triple Quadrupole (QQQ) scanning were performed using the Triple quadrupole linear ion TRAP Mass Spectrometer (QTRAP), AB4500 Q TRAP UPLC/MS/MS system equipped with ESI Turbo ion spray interface. Both positive and negative ion modes were controlled by the Analyst 1.6.3 software (AB Sciex). The ESI source operating parameters are as follows: ion source, turbo spray; Source temperature 550°C; Ion spray voltage (IS) 5500 V (positive ion mode)/-4500 V (negative ion mode); the ion source gas I (GSI), gas II (GSII), and curtain gas (CUR) are set to 50, 60, and 25.0 psi, respectively, and the collision-induced ionization parameter was set to high. The instrument was tuned and calibrated with 10 and 100 μmol/L polypropylene glycol solution in QQQ and LIT modes, respectively. The QQQ scan uses MRM mode with the collision gas (nitrogen) set to medium. Through further DP and CE optimization, the DP and CE of each MRM ion pair were determined. A specific set of MRM ion pairs was monitored during each period based on the metabolites elution times.

### 2.4 Statistical analysis

The metabolomic data were analyzed using multivariate statistical analysis. Heat maps were generated using the Complex Heatmap package in R software. Differential metabolites were annotated and presented using KEGG database (Kanehisa and Goto, 2000). The experimental data were processed using Excel 2021 software, followed by graphing using origin 2019 and Graphpad Prism8.3.3 softwares. Subsequently, the data were analyzed by ANOVA, correlation analysis and principal component analysis in SPSS 19.0 software.

## 3 Results and analyses

### 3.1 Quantitative analysis of total flavonoid content

**Figure 1:**
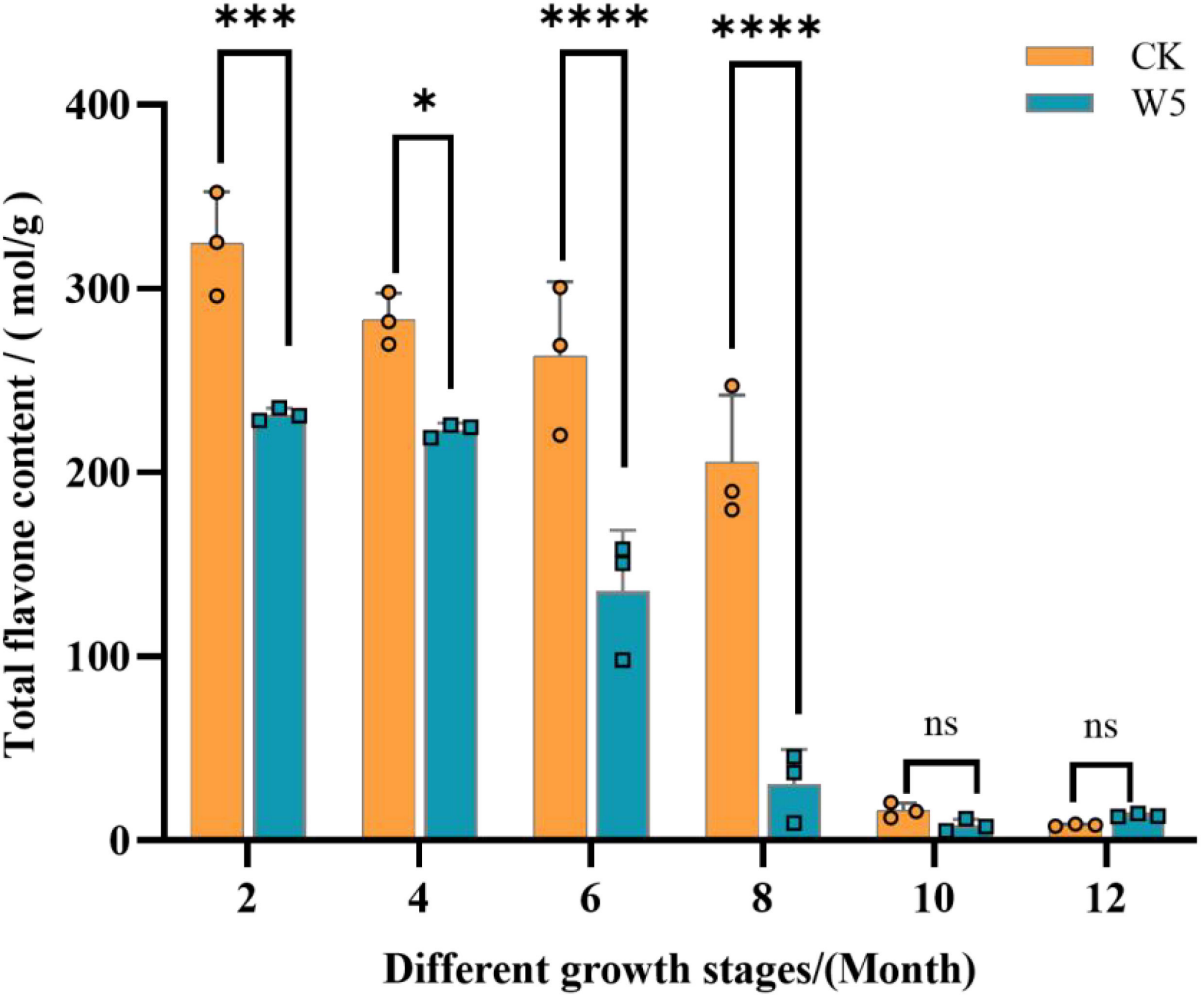
Dynamics of total flavonoid content in different varieties at different fruit ages. The bar chart illustrates the total flavonoid content in two coconut varieties, ‘CK’ denotes local coconut; ‘W5’ denotes ‘Wenye No. 5’; “2, 4, 6, 8, 10, 12” denotes the age of coconut fruits in different months. Error bars indicate the standard deviation of three replicates. Asterisks (*) denote significant differences in flavonoid content (p < 0.05) between the two varieties at the same fruit age. The mean values for asterisked bars are highlighted and annotated where applicable. ‘ns’ above the bar indicates no statistical significance, ****p<0.0001, ***p<0.001, *p<0.05, and ‘ns’ indicates p>0.05.

The results showed (**Figure 1**) that the total flavonoid content of both coconut varieties exhibited a gradual decline from 2 months to 12 months of age, with the highest content at 2 months of age. In terms of specific values, the total flavonoid content of the CK variety peaked at 325.00 mol/g at 2 months of age and reached a minimum value of 8.46 mol/g at 12 months of age, Similarly, the W5 variety peaked at 231.00 mol/g at 2 months of age and reached a minimum value of 13.6 mol/g at 12 months of age. Notably, the total flavonoid content of the CK variety declined significantly between 8 and 10 months of age, whereas the W5 varieties showed a significant decline between 6 and 8 months of age. Differences between the two breeds were significant for comparisons at 2, 4, 6, and 8 months of age, and non-significant for comparisons at 10 and 12 months of age.

### 3.2 Qualitative and quantitative analysis of total metabolites

**Figure 2:**
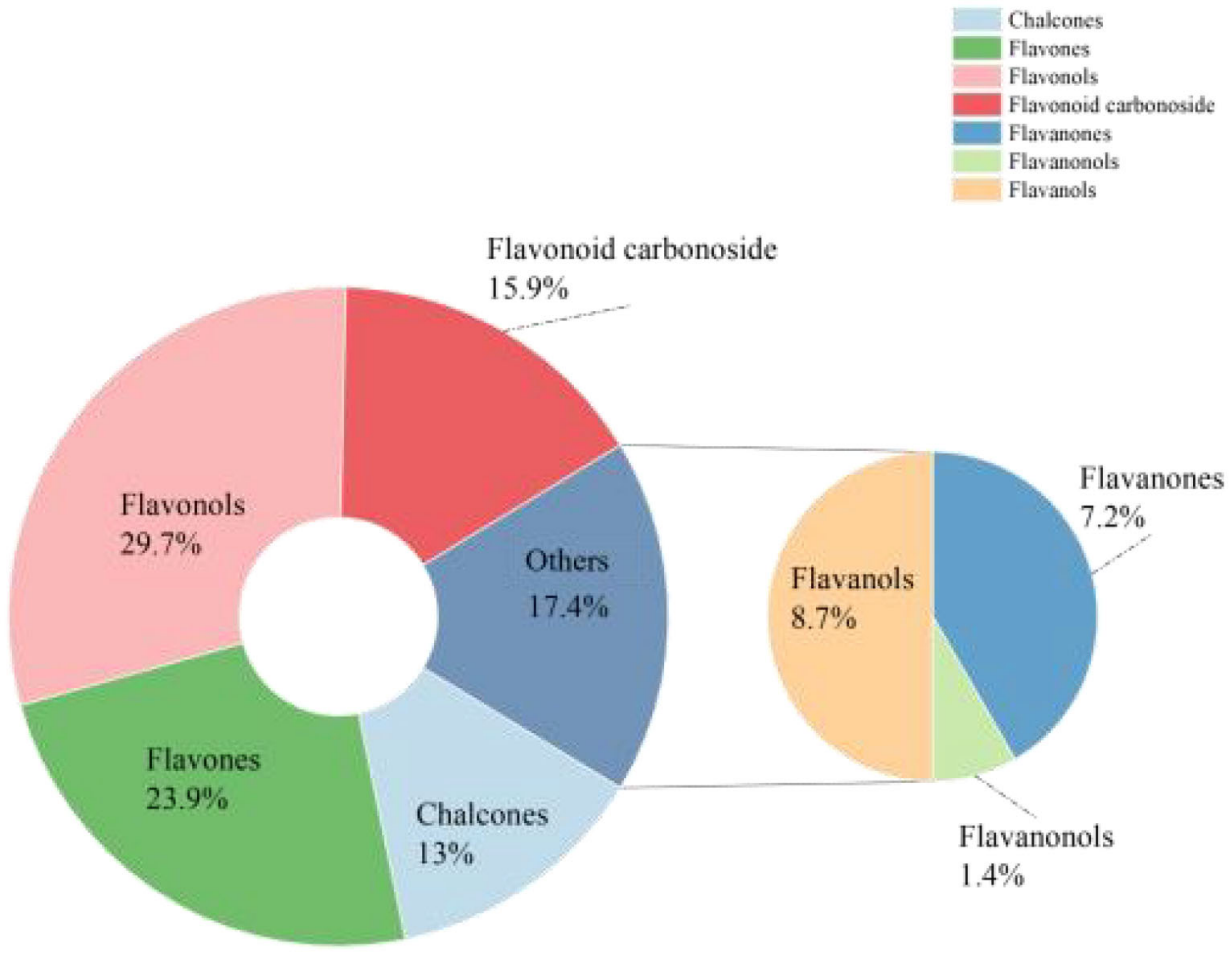
Pie chart showing types of flavonoid metabolites. The pie chart illustrates the distribution of different types of flavonoid metabolites. Each segment represents a specific class of flavonoids, with the size of each segment proportional to its relative abundance in the sample. The chart provides a visual summary of the composition of flavonoid metabolites.

**Figure 3:**
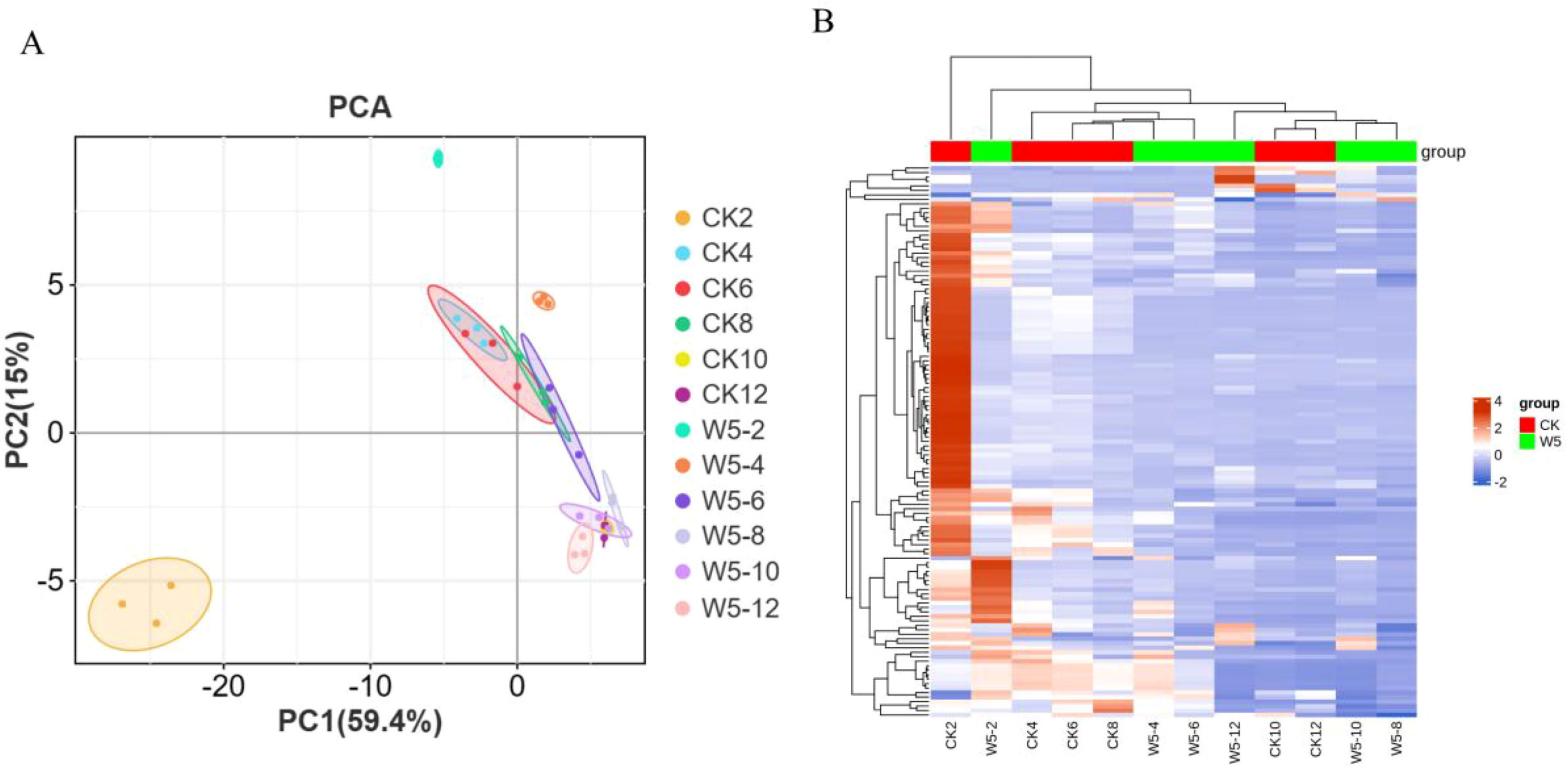
Quantitative and qualitative analysis of total flavonoid metabolites. **(A)** Principal Component Analysis (PCA) score plot illustrating the distribution of flavonoid metabolites based on their quantitative and qualitative profiles. Each point represents a sample, with the plot visualizing the separation and clustering of metabolites along principal components. **(B)** Heatmap showing the clustering of metabolites, with each row representing a different metabolite. The color gradient indicates abundance levels: red bars denote high abundance, while green bars indicate low abundance. The color key scale next to the heatmap provides a reference for interpreting the abundance levels.

To gain a deeper understanding of the flavonoid metabolic profiles of two coconut varieties, CK and W5, at different developmental stages, coconut water samples from both varieties were analyzed using LC-MS/MS-based metabolomics. Based on the local metabolic database, the samples were analyzed qualitatively and quantitatively by mass spectrometry, and the multi-reaction detection mode MRM metabolite multi-peak diagrams in the figure demonstrated the metabolites in the samples, and the mass spectrometry peak generations of different colors represented different metabolites, (**Supplementary Figures S1**, **S2**), in which the relative contents, integral values, and metabolite names corresponding to the chromatographic peaks of the flavonoid metabolites were shown in **Supplementary Table S1**. The results showed that a total of 123 flavonoid metabolites were identified, including 18 chalcones, 10 Flavanones, 2 Flavanonols, 33 Flavones, 41 Flavonols,22 Flavonoid carbonoside, and 12 Flavanols. (**Supplementary Table S1**; **Figure 2**). Unsupervised PCA analyses (**Figure 3A**), showed significant differences in metabolite composition between samples from different developmental periods. The PC2 axis revealed similar trends in flavonoid metabolite changes in CK and W5 across different harvesting periods. Furthermore, hierarchical clustering analysis (**Figure 3B**) revealed a specific clustering pattern of metabolite composition between CK and W5 at different developmental stages, with the highest accumulation of metabolites during the CK2 period and relatively low during the other periods.

### 3.3 Identification and screening of differential metabolites

**Figure 4:**
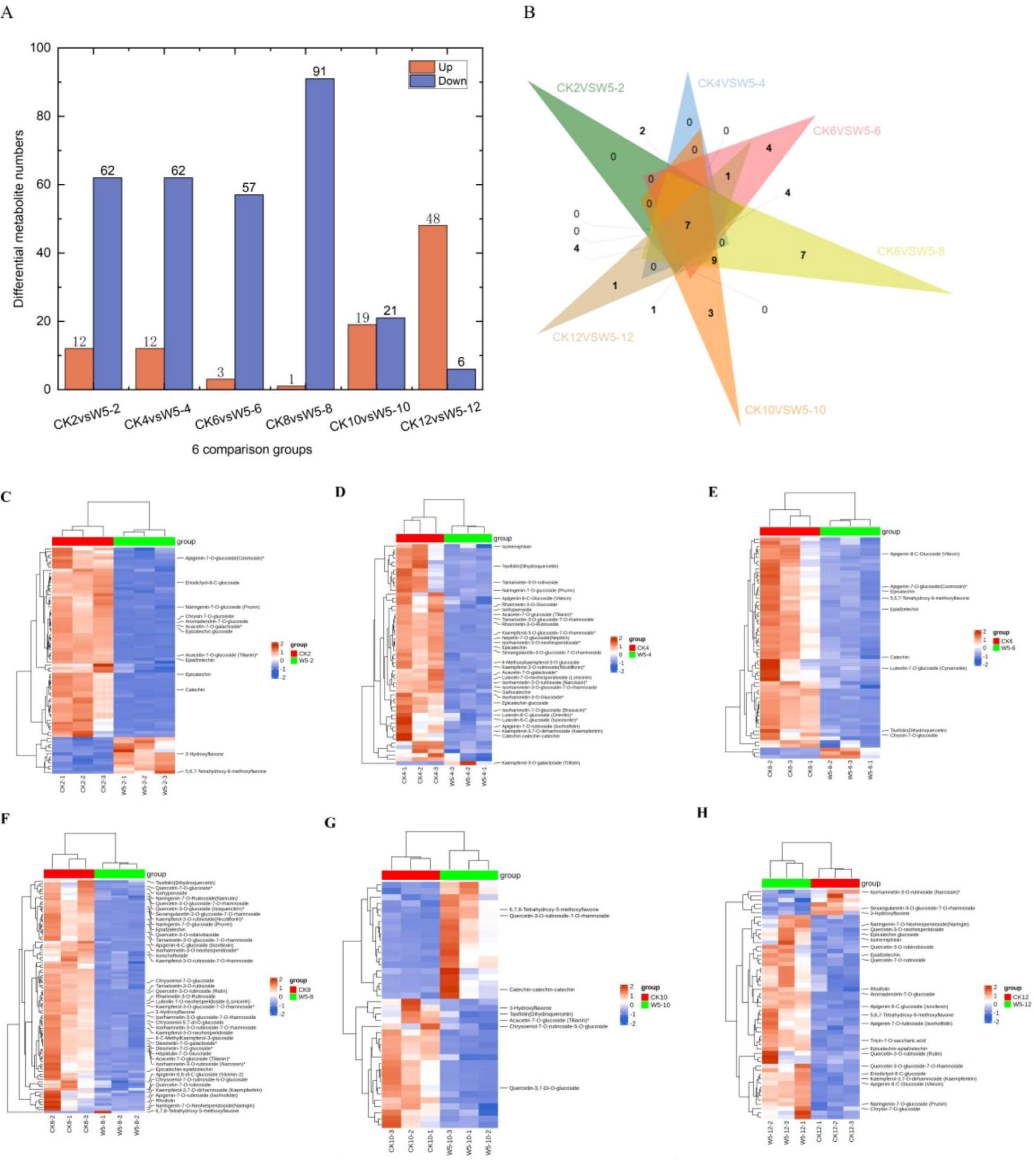
Differential metabolic analysis of two varieties, CK and W5, at different periods of fruit age. **(A)** Number of upward and downward adjustments in each comparison. **(B)** Venn of the number of differential metabolites in the six period comparisons. **(C–H)** denotes metabolite clustering analysis of samples from each period of CKvsW5.

Based on the criteria of fold-change ≥ 2 or ≤ 0.5 and VIP ≥ 1, we identified differential metabolites in CK and W5 fruits. The results showed that 38 common differential metabolites were identified (**Supplementary Table S2**), all of which were down-regulated. In CK2vsW5-2, 74 differential metabolites were identified, with 12 up-regulated and 62 down-regulated (**Supplementary Table S3**; **Figures 4A, C**). Similarly, in CK4vsW5-4, 74 differential metabolites were identified, with 12 up-regulated and 62 down-regulated (**Supplementary Table S4**; **Figures 4A, D**). In CK6vsW5-6 comparison, 60 differential metabolites were identified, with 3 up-regulated and 57 down-regulated (**Supplementary Table S5**; **Figures 4A, E**). In CK8vsW5-8, 92 differential metabolites were identified, with only 1 up-regulated and 91 down-regulated (**Supplementary Table S6**; **Figures 4A, F**). In CK10vsW5-10 comparison, 40 differential metabolites were identified, with 19 up-regulated and 21 down-regulated (**Supplementary Table S7**; **Figures 4A, G**). Finally, in CK12vsW5-12, 54 differential metabolites were found, with 48 up-regulated and 6 down-regulated (**Supplementary Table S8**; **Figures 4A, H**).

Across all comparison groups, seven common differential metabolites were identified (**Figure 4B**). These include catechin, epicatechin, Dihydroquercetin, rhamnetin-3-O-glucoside, epicatechin-epiafzelech, apigenin-7-O-rutinoside, and kaempferol-3,7-O-dirhamnoside (**Table 1**). Additionally, several differential metabolites specific to certain comparison groups were also identified. For instance, in the CK12vsW5-12 comparison, a unique differential metabolite Isohemiphloin was found. In CK10 vs W5-10 comparison, three unique differential metabolites were identified: eupatilin-7-O-glucoside,Vitexin-2’’-O-galactoside, Quercetin-3-O-rutinoside-7-O-rhamnoside. Furthermore, in the CK8 vs W5-8 comparison, seven unique differential metabolites were identified: Luteolin-8-C- arabinoside, Quercetin-4’-O-glucoside (Spiraeoside), Vitexin-2’’-O-rhamnoside, Apigenin-6,8-di-C-glucoside (Vicenin-2). These findings provide important clues for future insights into flavonoid metabolite changes in coconut fruits at different developmental stages.

**Table 1:** Exhaustive information on common differential metabolites for six periods of fruit age in CKvsW5.

Index	Q1 (Da)	Molecular Weight (Da)	Formula	Ionization model	Class	Compounds
MWSHY0167	291.00	290.00	C_15_H_14_O_6_	[M+H]+	Flavanols	Catechin
MWSHY0174	291.09	290.08	C_15_H_14_O_6_	[M+H]+	Flavanols	Epicatechin
mws0044	303.05	304.06	C_15_H_12_O_7_	[M-H]-	Flavanonols	Taxifolin(Dihydroquercetin)
Lmjp002906	479.12	478.11	C_22_H_22_O_12_	[M+H]+	Flavonols	Rhamnetin-3-O-Glucoside
pmb3114	561.14	562.15	C_30_H_26_O_11_	[M-H]-	Flavanols	Epicatechin-epiafzelechin
pme0368	579.17	578.16	C_27_H_30_O_14_	[M+H]+	Flavones	Apigenin-7-O-rutinoside
pme2493	579.17	578.16	C_27_H_30_O_14_	[M+H]+	Flavonols	Kaempferol-3,7-O-dirhamnoside

### 3.4 Orthogonal Partial Least Squares Discriminant Analysis of Differential Metabolite Groupings

**Figure 5:**
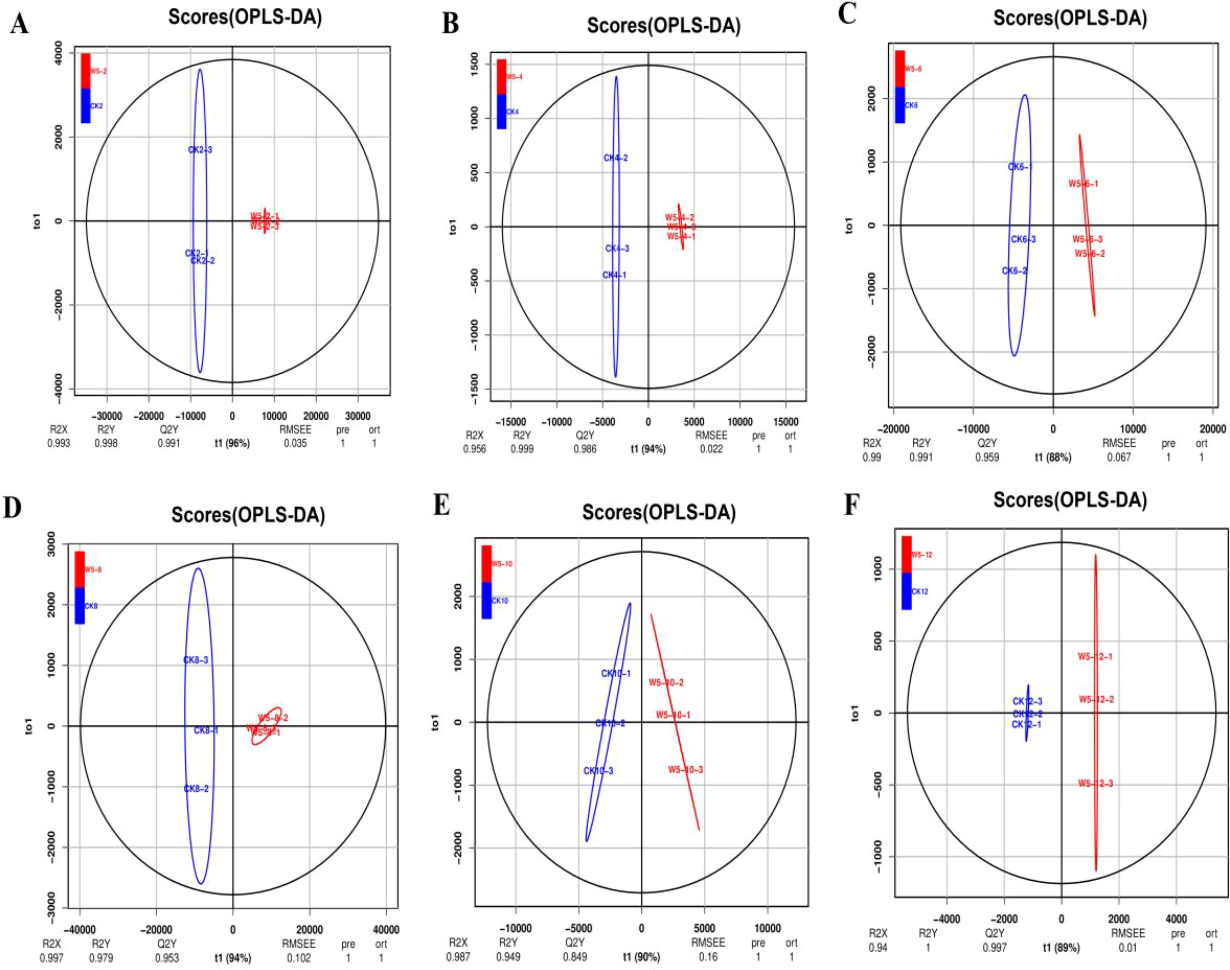
OPLS-DA model plots of differential flavonoid metabolite analysis across different fruit ages. **(A-F)** Show the OPLS-DA model plots for the following comparisons: **(A)** CK2 vs. W5-2, **(B)** CK4 vs. W5-4, **(C)** CK6 vs. W5-6, **(D)** CK8 vs. W5-8, **(E)** CK10 vs. W5-10, **(F)** CK12 vs. W5-12. The plots display the separation and classification of flavonoid metabolites based on orthogonal signal correction and partial least squares discriminant analysis (OPLS-DA) for various fruit maturation stages.

**Figure 6:**
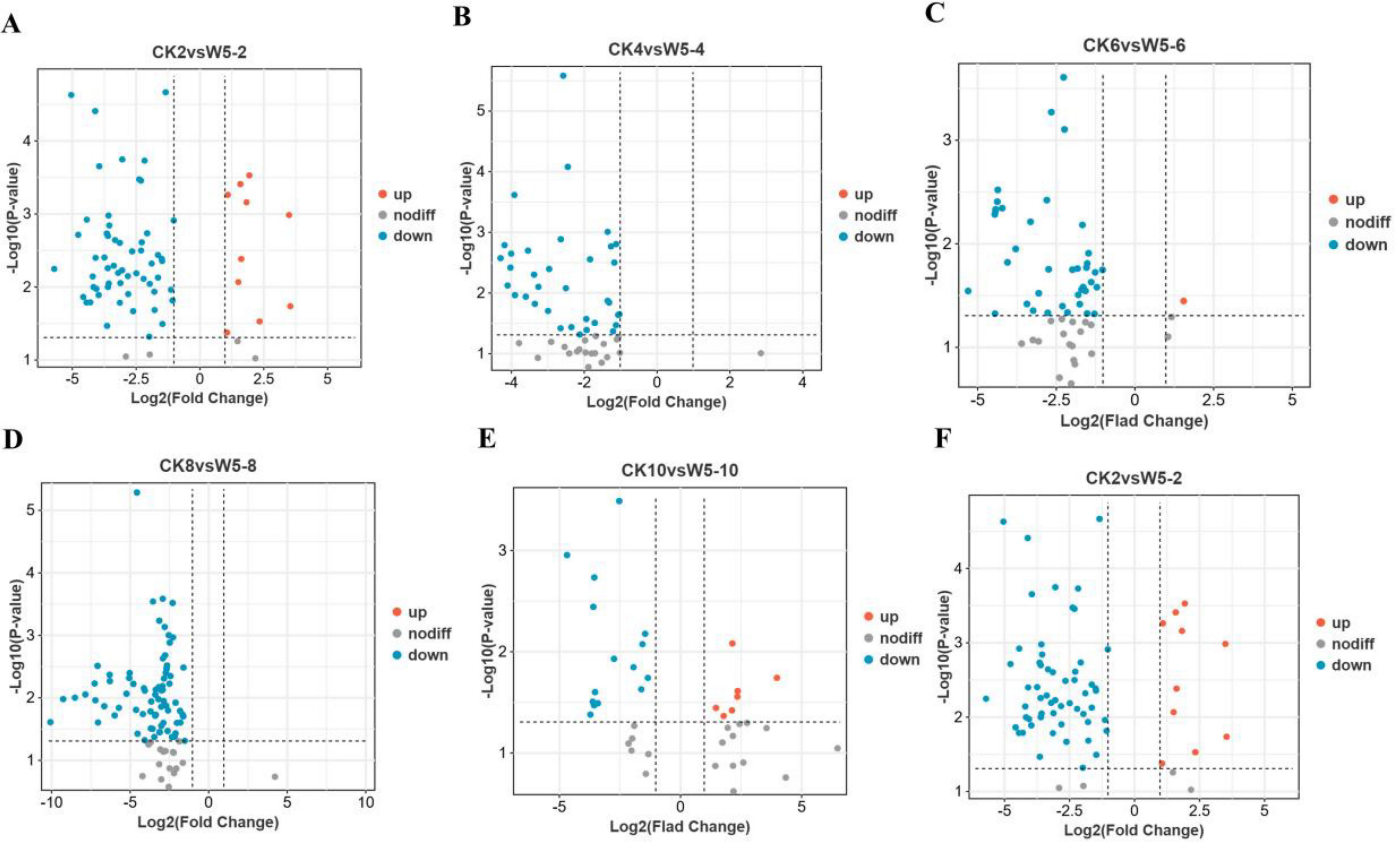
Volcano plots of metabolites differing by fruit age in different coconuts. **(A-F)** Present volcano plots comparing CK and W5 for various fruit ages. Each point represents a metabolite, with the horizontal axis showing the logarithm of the fold change between the two sample varieties (Log2 fold change) and the vertical axis representing the significance of the variable (VIP). Larger absolute values on the horizontal axis indicate greater significance of the difference. Blue dots denote down-regulated metabolites, red dots represent up-regulated metabolites, and gray dots indicate metabolites with significant changes.

Initial screening was performed based on VIP values (variable importance in projection) obtained using the OPLS-DA method, with screening criteria set at a fold change ≥2 or ≤0.5 and VIP ≥1. According to the OPLS-DA model (**Figures 5A–F**), a significant separation was observed between CK2, CK4, CK6, W5-6, CK8, CK10, W5-6, W5-10, and W5-12, whereas the separation between W5-2, W5-4, and W5-8 was insignificant. This suggests that metabolism varied more significantly among varieties at different fruit ages. The 123 differential metabolites were further analyzed, and the results of volcano plot analysis (**Figures 6A–F**) showed that 74, 60, 92, 40, and 54 differential metabolites were identified for CK2 vs W5-2; CK4 vs W5-4; CK8 vs W5-8; CK6 vs W5-6; CK8 vs W5-8; and CK12 vs W5-12, respectively (**Supplementary Table S9**), This analysis revealed the up- and down-regulation trends for each comparison, and the number of up- and down-regulated metabolites differed across comparison groups. For instance, in the CK2 vs. W5-2 comparison, 12 differential metabolites were up-regulated and 62 differential metabolites were down-regulated, whereas in the CK12 vs. W5-12 comparison, 48 differential metabolites were up-regulated and 6 differential metabolites were down-regulated.

### 3.5 KEGG functional annotation and enrichment analysis of differentially expressed metabolites

**Figure 7:**
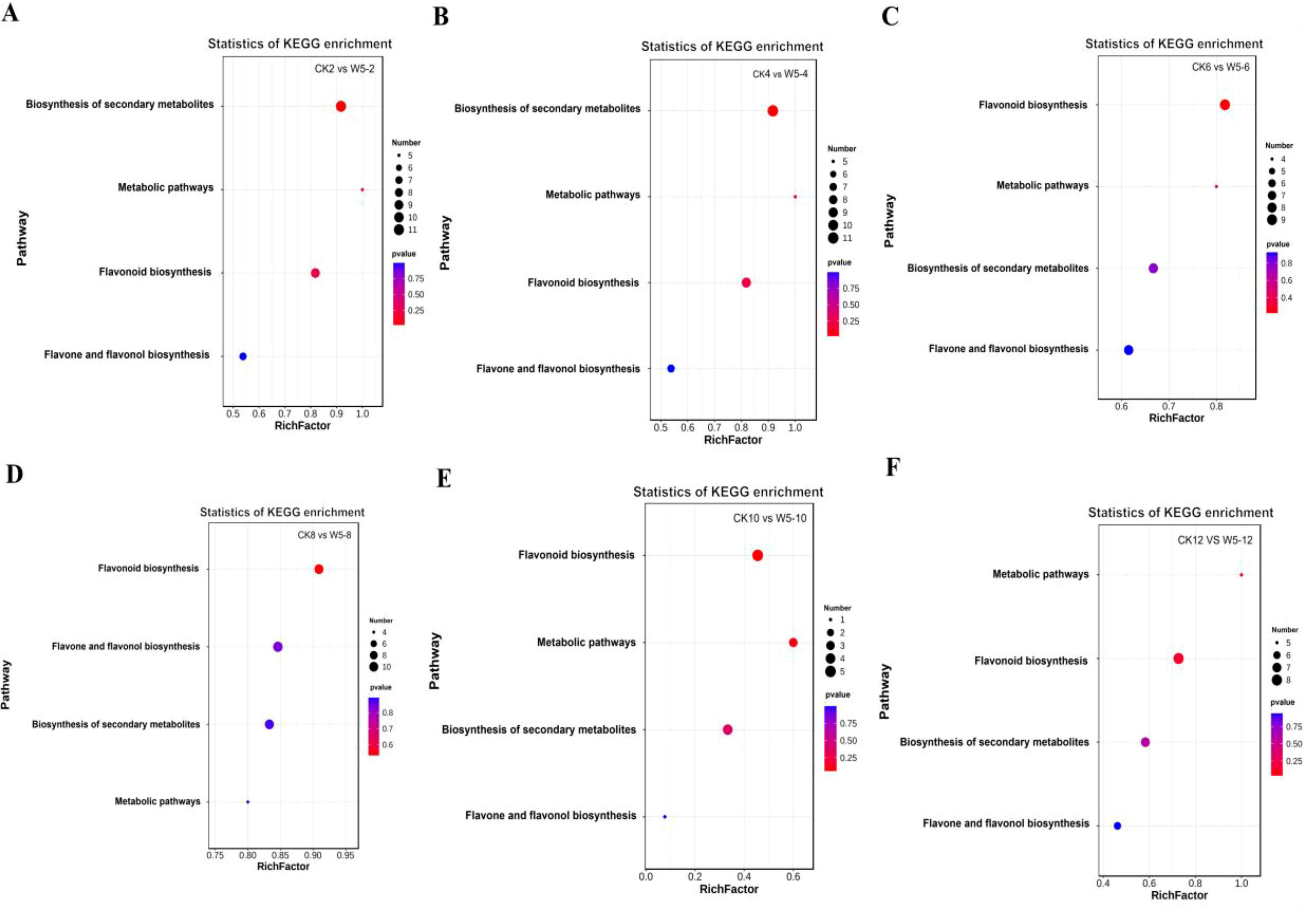
Enrichment analysis of differential flavonoid metabolites in six comparison groups. **(A-F)** Show the classification of differential metabolites in CK vs. W5 across different comparisons. Bubble size represents the number of metabolites significantly enriched in the pathway, with larger bubbles indicating a greater number of enriched metabolites.

**Figure 8:**
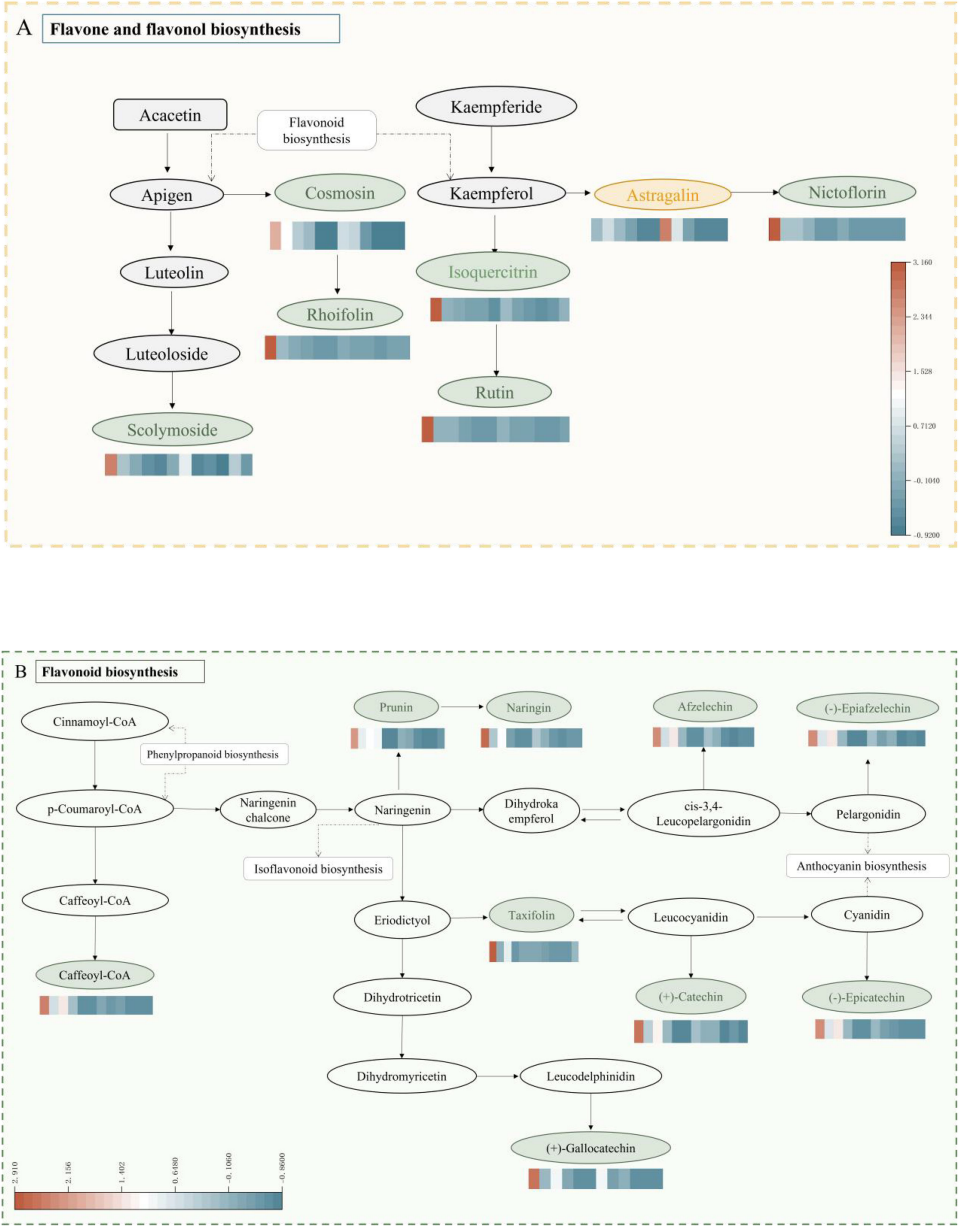
Enriched KEGG pathways of differentially expressed metabolites. **(A)** indicates flavonoid and flavonol biometabolic pathways, **(B)** indicates flavonoid biometabolic pathways. The color gradient represents the relative abundance of each flavonoid. Typically, warmer colors (red, orange) indicate higher levels, while cooler colors (blue, green) indicate lower levels.

The functional annotation of differentially expressed metabolites and KEGG pathway enrichment analysis are shown in **Figures 7A-F**. The differentially expressed metabolites were primarily enriched in four metabolic pathways: the metabolic pathway (ko 01100), the flavonoid biosynthesis pathway (ko 00941), the biosynthesis of secondary metabolites pathway (ko 01110), and the biosynthesis pathway of flavonoids and flavonols (ko 00944). In the present study, it was found that during fruit ripening, the flavonoid metabolites remained abundant, although their accumulation level decreased. Differential expressed metabolites were mainly enriched in the biosynthetic pathways of flavonoids and flavonols, which is considered the main pathway for flavonoid synthesis in coconut plants (**Figure 8**). Further analysis of the 38 differential metabolites annotated to these pathways revealed a diverse range of compounds including chalcones, Flavanones, Flavanonols, Flavones, Flavonols, Flavonoid carbonoside, and Flavanols. (**Supplementary Table S2**).

### 3.6 Comprehensive comparison of flavonoid differential metabolites and analysis of characterized metabolites

The accumulation levels of 38 differential metabolites were analyzed across different harvest periods, and the described differentials were annotated with distinct flavonoid metabolic pathways CK and W5 fruits, as well as these metabolic networks (**Figure 8**). The majority of these metabolites were primarily enriched in the biosynthesis pathway of flavonoids and flavonols and the flavonoid biosynthesis pathway. The results showed that in the flavonoid and flavonol biosynthesis pathway, this study detected low expression of metabolites such as scolymoside, rutin, and nictoflorin during fruit development, whereas metabolites like kaempferol 3-O-glucoside were highly expressed. In the flavonoid biosynthesis pathway, metabolites such as (+)-catechin, (-)-epicatechin, gallocatechin, and (-)-epiafatechin were found to be lowly expressed during fruit development.

## 4 Discussion

Flavonoid is one of the important compounds in coconut water, which has an important effect on the growth and development of coconut. The change trend and metabolic mechanism of flavonoid in coconut water at different developmental stages are still unclear. In this study, the related problems were studied by metabonomics method. By examining the flavonoid content and metabolic properties of two coconut varieties (CK and W5) at different stages of fruit development, the results revealed that the total flavonoid content of both varieties exhibited a significant decline with fruit maturation, with the highest content observed at the age of fruit in 2 months. The total flavonoid content of CK varieties was not only numerically higher than that of W5, but also displayed more significant variation (**Figure 1**), suggesting that CK varieties may possess a more active metabolic mechanism for flavonoid synthesis and regulation, whereas W5 varieties exhibited a more stable metabolic profile. This finding highlighted essential differences in flavonoid metabolism among coconut varieties. Further analysis showed that the total flavonoid content decreased sharply during specific stages of coconut fruit development, such as between 8 and 10 months of age (**Figure 1**). This phenomenon may be attributed to the slowing down of flavonoid synthesis rates during fruit ripening, the changes in internal chemical composition, and the combination of flavonoids with other compounds (such as free glycoside elements and glycosides) to form more stable and water-soluble glycosidic compounds (such as flavonols, flavonoid glycosides). These glycosidic compounds play a crucial role in plant growth and development (Nabavi et al., 2020; Ojeda et al., 2002; Ferreyra et al., 2007). The metabolic trends of flavonoid compounds in plants have been reported to be diverse. For example, similar decreasing trends in total flavonoid content have been observed in grapes, strawberries, and pecans (Awad et al., 2017; Ferreyra et al., 2007; Jia et al., 2018), whereas contrasting findings have been reported in green beans and mangoes (Huang et al., 2023; Gan et al., 2016; Lu et al., 2019), which highlights the complexity and diversity of flavonoid metabolism across the plant kingdom. As an important secondary metabolite in plants, flavonoids have a more intricate synthesis and metabolism process (Liu et al., 2021). Research has shown that flavonoid biosynthesis in plants is not only tissue-specific, but also regulated by the plant’s developmental stage and influenced by environmental factors (Yang et al., 2018; Shi et al., 2022). Changes in plant chemical composition and bioactivity are strongly correlated with changes in different metabolic stages and climatic factors, such as temperature, soil moisture and rainfall (Yang et al., 2016; Prinsloo and Nogemane, 2018). Drought, high temperature and light exposure affect flavonoid and phenolic content in plants (Carvalho et al., 2010; Zheng et al., 2012; Liu et al., 2011). For instance, high temperatures (30-40°C) inhibit flavonoid biosynthesis, gene expression and enzyme activity (Dela et al., 2003). The sharp decline in flavonoid content between 8 and 10 months of age in the present study may be attributed to the high temperatures during this period. In addition to environmental factors, the differences in the synthesis of different species of flavonoids may also be related to the metabolic pathway, the synthesis of flavonoids involves the metabolic pathway of phenylpropanoid compounds, which involves the catalysis of several enzymes (PAL, C4H, 4CL), which catalyze the deamination of the aromatic amino acid phenylalanine (Phe) to produce coumaric acid (by PAL), then oxidized to 4-coumaric acid (by C4H). Through the addition of coenzyme A, the final activation into p-acyl-CoA (4CL), after a series of complex reactions, the formation of a series of phenolic substances and other secondary metabolites. In addition to environmental factors, differences in the synthesis of flavonoids in different species may be related to metabolic pathways. Flavonoid synthesis involves the metabolic pathway of phenylpropanoids, which involves the catalysis of several enzymes (PAL, C4H, 4CL), These enzymes catalyze the deamination of the aromatic amino acid phenylalanine (Phe) to form coumaric acid (by PAL), which is then oxidized to 4-coumaric acid (by C4H). Then, a series of secondary metabolites such as phenolics are formed through the addition of coenzyme A (CoA), which is finally activated into p - acyl- CoA (4CL), after a series of complex reactions (Nabavi et al., 2020). Flavonoids may also undergo a series of modification (glycosylation, methylation) and translocation processes after synthesis, which also affect the final content and distribution of flavonoids. The Arabidopsis UDP glycosyltransferases, UGT79B2 and UGT79B3, function as anthocyanin rhamnosyltransferases that confer abiotic stress tolerance by regulating anthocyanin accumulation (Li et al., 2017), There may be differences in the activity of these enzymes and substrate availability in different species, leading to differences in the rate of flavonoid synthesis and products. On the other hand, genetic factors also influence the differences in flavonoid content among different varieties, and the expression of genes involved in the flavonoid synthesis pathway varies among different varieties of plants. These genes include genes encoding flavonoid synthases (CHS, CHI, F3H) and transcription factors that regulate the expression of these genes. Mutants of several pro-flavonoid compound synthesis key enzymes corresponding to the key genes CHS, CHI, F3′H, and DFR were isolated in Arabidopsis thaliana, which play an important role in the synthesis of plant flavonoid compounds (Shen et al., 2022); In tobacco, both transcription factors NtMYB12a and NtMYB12b are involved in regulating flavonoid biosynthesis; and in tomato, the transcription factor WD40 protein SlAN11 is coordinated with bHLH and MYB proteins to regulate flavonoid biosynthesis and seed dormancy (Gao et al., 2018); Overexpression of the transcription factor MdWRKY11 in apple promoted the expression of F3H, FLS, DFR, anthocyanin synthase (ANS), and UFGT, which contributed to the increased accumulation of flavonoids and anthocyanins in apple healing tissues (Wang et al., 2018). CsMYB6A and CsUGT72AM1, key genes identified from tea tree, regulate the accumulation of anthocyanins and flavonols in purple leaf tea (He et al., 2018).Differences in gene expression directly lead to differences in the rate and products of flavonoid synthesis, thus affecting the flavonoid content in different varieties. It is hypothesized that the decrease in flavonoid content in this study is related to the conversion of other substances, environmental factors, metabolic pathways and genetic factors.

The flavonoid species and their metabolic properties in coconut water were systematically analyzed by UPLC-MS/MS technique and metabolomics approach, resulting in the detection of a total of 123 flavonoid compounds. The results of PCA and cluster analyses further emphasized this difference, indicating that metabolite accumulation was significantly higher in the CK variety compared to the other periods and in W5 (**Figure 3**). In the screening of differential metabolites, we identified a series of metabolites that differed significantly between CK and W5 varieties, including catechins and epicatechins, which play key roles in flavonoid biosynthesis. There are four common catechins: (+)-catechin, (-)-catechin, (+)-epicatechin, and (-)-epicatechin. Catechin biosynthesis involves the general phenylpropane pathway, which branches out into specific pathways of flavonoid biosynthesis (Xiong et al., 2013; Yu et al., 2022). Numerous studies have demonstrated that low concentrations of catechins have hormonal effects, promote, and enhance resistance, whereas high concentrations of catechins may be inhibitory (Hong et al., 2015; Prithiviraj et al., 2007; Ahammed et al., 2018). Additionally, low doses of (±) - catechins have been shown to elicit a moderate increase in ROS accumulation in the meristematic tissues of treated plants, which may trigger cell wall loosening, thereby promoting cell division and cell expansion for growth (Ahammed et al., 2023). In this study, we found that metabolites such as (+)-catechin and (-)-epicatechin were detected in the flavonoid biosynthesis pathway of W5 compared to CK and showed a significant down-regulation trend. Furthermore, gallocatechin and (-)-epiafatechin also exhibited significant down-regulation trends during fruit development (**Figure 8**). Based on these findings, it was inferred that W5 grows and develops better relative to CK and may accumulate more metabolites such as amino acids, proteins and sugars.

Flavonoids from coconut water species have been found to possess medicinal value, based on the results of KEGG functional annotation and enrichment analyses, which showed that flavonoids and flavonols have distinctly different distributions in the two species, with CK containing a higher abundance of flavonoids, while W5 up-regulated the accumulation of metabolites, particularly kaempferol-3-O-glucoside (**Figure 8**). Notably, Kaempferol-3-O-glucoside has been reported to exhibit a range of biological activities, including antimicrobial, anti-inflammatory, neuro- and cardioprotective, antitumour and antidiabetic properties (Riaz et al., 2018). Additionally, it has been shown to inhibit intestinal α-glucosidase and α–amylase activities (Hong et al., 2013), may act as a potential insulin secretagogue, contributing to glucose homeostasis (Rey et al., 2019). The study showed low expression of several differential metabolites such as catechin, saccharin chalcone, lonicera glycosides, rutin and other metabolites. Notably, the study showed that the expression of catechin changed noticeably, whereas the levels of other flavonoid metabolites remained relatively stable. These findings suggest that kaempferol-3-O-glucoside, and catechin may be a class of differential substances characterized in CK and W5. Differential metabolites were mainly concentrated in the flavonoid and flavonol biosynthesis pathways (**Figure 7**), highlighting the importance of flavonoids in coconut fruit metabolism and implying that they may play a role in physiological functions and stress responses in the fruit.

## 5 Conclusion

The present study compared the changes in flavonoid metabolites in coconut water from CK and W5 varieties at different stages of growth. The results showed that t the flavonoid content decreased significantly with fruit maturation, with a peak at 2 months of age and then gradually decreased. The flavonoid content of CK varieties was higher than that of W5 and varied more drastically, indicating fundamental differences in flavonoid synthesis and regulation between the two varieties. KEGG enrichment analyses showed that differential metabolites were mainly enriched in the flavonoid biosynthesis pathway (ko 00941), the flavonoid and flavonol biosynthesis pathway (ko 00944). Furthermore, the accumulation of key metabolites, such as kaempferol-3-O-glucoside and catechin, varied according to cultivar and developmental stage. This study not only enhanced our understanding of differential metabolism of flavonoids in coconut water, but also provided valuable insights into the study of flavonoid metabolic pathways and functions, which can contribute to the improvement of coconut varieties and quality enhancement. However, the study was limited by the sample size and inadequate analysis of environmental factors, and the specific mechanisms of flavonoids in coconut development require further in-depth exploration. Future studies should focus on these aspects to fully elucidate the complexities of flavonoid metabolism in coconut water.

## Data Availability

The datasets presented in this study can be found in online repositories. The names of the repository/repositories and accession number(s) can be found in the article/**Supplementary Material**.
