# Supplementary figures and images for: Dynamics of flavonoid metabolites in coconut water based on metabolomics perspective
Source: Front Plant Sci. 2024 Oct 7;15:1468858. doi: 10.3389/fpls.2024.1468858 (PMC11491327; doi:10.3389/fpls.2024.1468858)

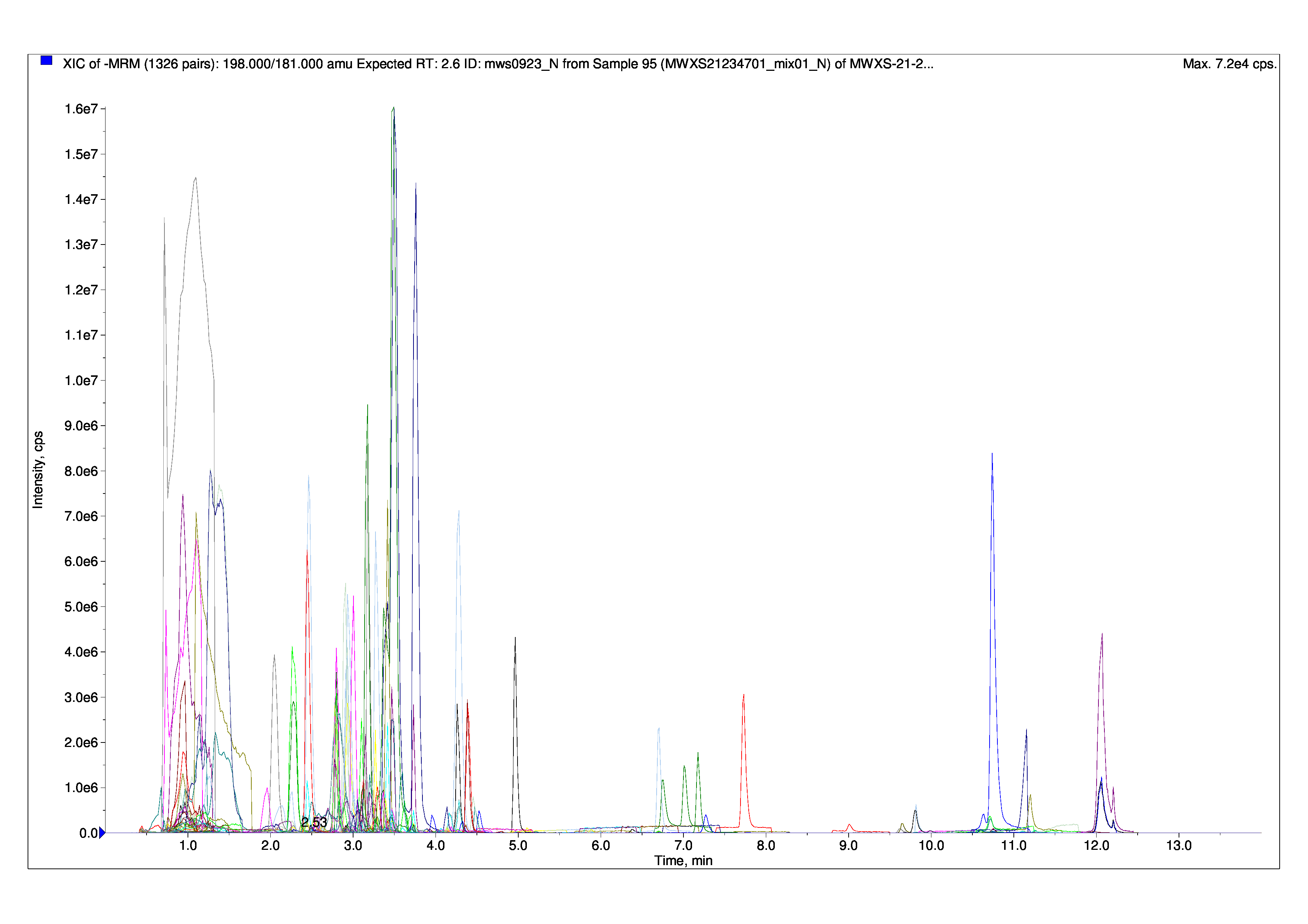

Supplement: Supplementary file 10 [file Image1.png]

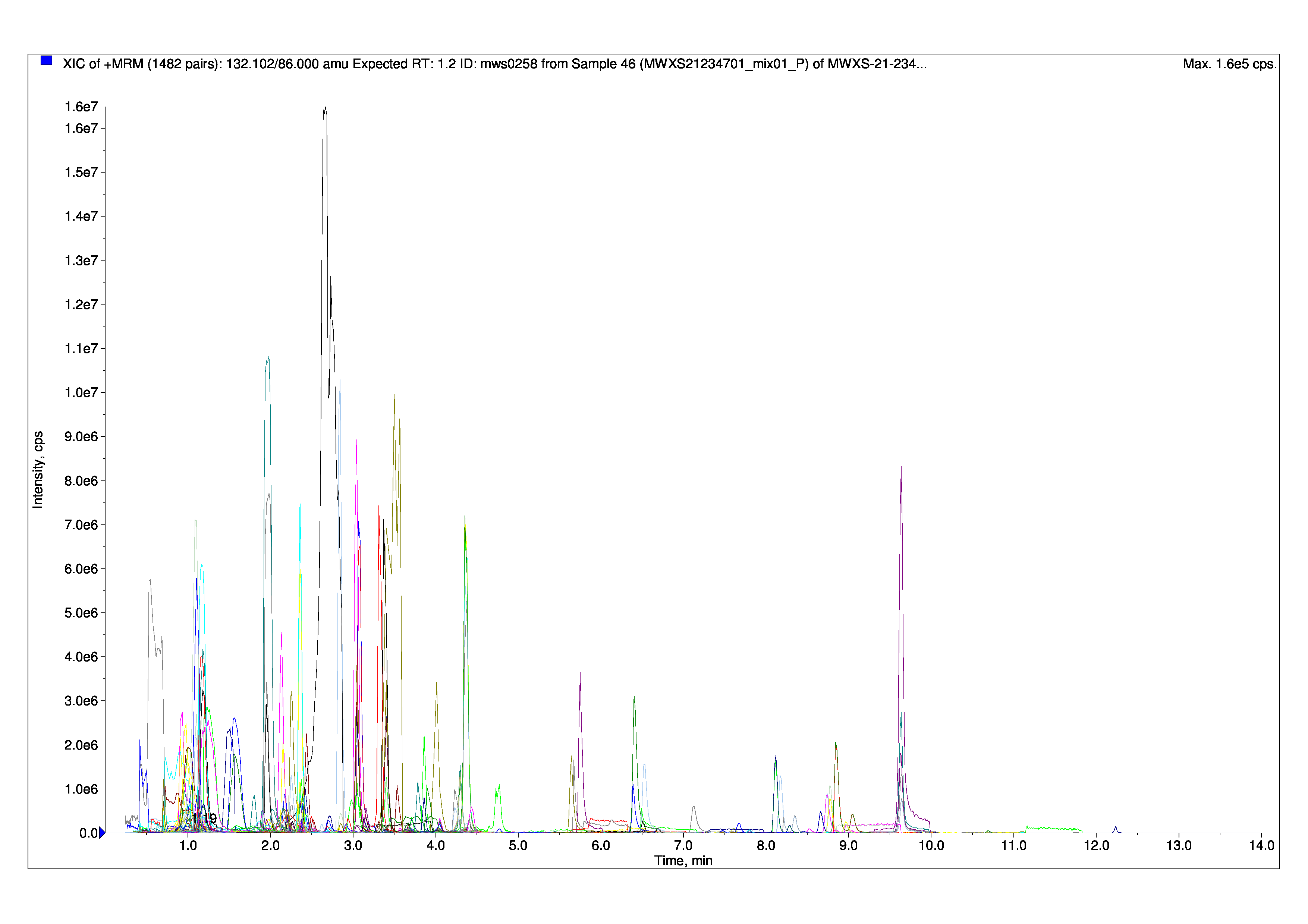

Supplement: Supplementary file 11 [file Image2.png]
